# Effect of fermentation and dry roasting on the nutritional quality and sensory attributes of quinoa

**DOI:** 10.1002/fsn3.1247

**Published:** 2019-11-06

**Authors:** Vanesa Castro‐Alba, Claudia Eliana Lazarte, Daysi Perez‐Rea, Ann‐Sofie Sandberg, Nils‐Gunnar Carlsson, Annette Almgren, Björn Bergenståhl, Yvonne Granfeldt

**Affiliations:** ^1^ Department of Food Technology, Engineering and Nutrition Lund University Lund Sweden; ^2^ Food and Natural Products Center San Simón University Cochabamba Bolivia; ^3^ Department of Chemical and Biological Engineering Food Science Chalmers University of Technology Gothenburg Sweden

**Keywords:** dry roasting, fermentation, minerals, phytate degradation, quinoa, sensory attributes

## Abstract

**Background:**

Quinoa is a pseudocereal with relatively high content of proteins and minerals that also contains mineral inhibitors such as phytate. The aim of the present study was to evaluate lactic acid fermentation and dry roasting on the nutritional quality and sensory attributes of quinoa. Various processes were evaluated, and quinoa grains were dry‐roasted, milled, and fermented, either with or without the addition of wheat phytase or activated quinoa phytase (added as back‐slop starter), for 10 hr. In other processes, raw quinoa flour was fermented for 10 hr or 4 hr and dry‐roasted. Hedonic sensory evaluation was then performed to evaluate the acceptability of the fermented flours prepared as porridges.

**Results:**

The combined dry roasting and fermentation processes significantly (*p* < .05) degraded phytate between 30% and 73% from initial content. The most effective process was fermentation of raw quinoa flour followed by dry roasting, which improved the estimated zinc and iron bioavailability. Particularly, estimated zinc bioavailability improved from low (Phy:Zn 25.4, Phy·Zn:Ca 295) to moderate (Phy:Zn 7.14, Phy·Zn:Ca 81.5). Phytate degradation was mainly attributed to the activation of endogenous phytase during fermentation. Dry roasting was effective in improving the sensory attributes of the fermented quinoa flour. Porridge made with raw quinoa flour fermented for 4 hr and dry‐roasted was more favorable to overall acceptability than that which was fermented for 10 hr and dry‐roasted.

**Conclusion:**

Fermentation of quinoa flour for 4 hr followed by dry roasting was successful in improving both nutritional and sensory attributes of the final product.

## Graphical abstract

The combined dry roasting and fermentation processes degraded phytate in quinoa between 30% and 73% from initial content. The most effective process was fermentation of raw quinoa flour followed by dry roasting, which improved the estimated zinc and iron bioavailability. Phytate degradation was mainly attributed to the activation of endogenous phytase during fermentation. Dry roasting was effective in improving the sensory attributes of the fermented quinoa flour. Fermentation of quinoa flour for 4 hr followed by dry roasting was successful in improving both nutritional and sensory attributes of the final product.

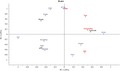

## 1 INTRODUCTION

Quinoa (*Chenopodium quinoa* Willd), a pseudocereal originated in the Andean region of South America, is a food commonly consumed in Bolivia. It has a higher protein content and quality than conventional cereals (Békés, Schoenlechner, & Tömösközi, 2017). The iron, zinc, and calcium contents are also higher than in conventional cereals (Lazarte, Carlsson, Almgren, Sandberg, & Granfeldt, 2015; Reguera & Haros, 2017). Quinoa, however, contains antinutritional compounds, such as saponins, and mineral absorption inhibitors such as phytate (myo‐inositol hexakisphosphate) and polyphenols (Lazarte, Carlsson, et al., 2015; Repo‐Carrasco‐Valencia, Hellström, Pihlava, & Mattila, 2010; Ruales & Nair, 1993). Phytate is the main storage form of phosphorus in mature grains, but impairs the absorption of iron, zinc, and calcium forming insoluble complexes with these nutrients (Reddy & Sathe, 2001; Schlemmer, Frolich, Prieto, & Grases, 2009). The phytate effect on the impairment of mineral absorption follows a dose‐dependent response; thus, the following molar ratios have been suggested as indicators of mineral bioavailability: phytate:iron (Phy:Fe) <1; phytate:zinc (Phy:Zn) <15; phytate·calcium:zinc (Phy·Ca:Zn) <200; and phytate:calcium (Phy:Ca) <0.17 (Brown et al., 2004; Gibson, Bailey, Gibbs, & Ferguson, 2010; Hurrell & Egli, 2010). Phytate can be hydrolyzed to lower myo‐inositol phosphates and free phosphate by the enzyme phytase, which is present endogenously in raw grains. Optimal phytase activity depends on pH, temperature, moisture, and type of food (Sanz‐Penella, Tamayo‐Ramos, & Haros, 2012). Wheat and rye, for example, are grains with high phytase activity; quinoa possesses lower phytase activity than wheat, but higher than oat (Egli, Davidsson, Juillerat, Barclay, & Hurrell, 2002). In order to achieve higher phytate hydrolysis, exogenous phytase from plant origin (e.g., wheat phytase) or microbial origin (e.g., lactic acid bacteria) can be added during the processing of foods. Alternatively, processing techniques such as soaking, early‐stage thermal treatment, germination, and fermentation can be applied to activate endogenous phytase (Sandberg & Andlid, 2002). Conversely, phytase activity can be reduced or inactivated completely by other processing techniques such as dehulling or thermal treatments at high temperatures (Greiner & Konietzny, 1998).

Lactic acid fermentation has previously been shown to reduce the phytate content in several roots (cassava; Lazarte, Vargas, & and, 2015) and cereals (millet and quinoa) (Sharma & Kapoor, 1996; Valencia, Svanberg, Sandberg, & Ruales, 1999). The mechanism involves the activation of endogenous phytase (Sandberg & Andlid, 2002). In addition, during lactic acid fermentation compounds that confer pleasant or unpleasant flavor can be produced and thus modify the sensory attributes of food (Di Renzo, Reale, Boscaino, & Messia, 2018; Hammes et al., 2005). Dry roasting is an important process for modifying the sensory properties and color of foods. The changes in these properties are mainly due to the Maillard reaction, which occurs between amino acids and reducing sugars, producing different flavor and color compounds, as well as caramelization reactions between sugars (Brady, Ho, Rosen, Sang, & Karwe, 2007; Fayle, 2002). There is a lack of information on the combined effects of fermentation and dry roasting on phytate degradation and on the particular sensory properties of the quinoa flour.

The aim of the present study was to achieve a better nutritional quality and sensory properties of quinoa through processes of fermentation and dry roasting.

## 2 MATERIALS AND METHODS

### 2.1 Materials

Quinoa grains of Bolivian origin were purchased from Productos Alimenticios Andes Trópico, a commercial supplier, in Cochabamba, Bolivia, and at ICA Supermarket in Lund, Sweden, in 2017. Each batch was mixed and separated into portions of 500 g, vacuum‐packed, and stored under refrigeration (4°C) and in darkness to avoid mold growth. *Lactobacillus plantarum* 299v^®^ (ProbiMage, Sweden) was used as a starter culture for the fermentation of quinoa. Wheat phytase (Enzyme Commission number 3.1.3.26, activity ≥ 0.01 unit/mg solid, Sigma‐Aldrich, St. Louis) was used as an exogenous source of phytase.

### 2.2 Fermentation processes of quinoa flour

Quinoa is not commonly fermented in Bolivia. However, purple maize flour is commonly fermented prior to preparing a thick porridge that is consumed during breakfast or evening snacks. The “traditional fermentation process” includes preparation of a mixture of purple maize flour and water, which is spontaneously fermented usually for 24 hr to 48 hr at room temperature (25°C – 35°C). In our previous study (Castro‐Alba et al., 2019), this process of spontaneous fermentation was applied to quinoa and compared to fermentation with *L. plantarum*, the latest resulted in a more controlled fermentation process, and therefore, it was used in the present paper as described below.

**Figure 1:**
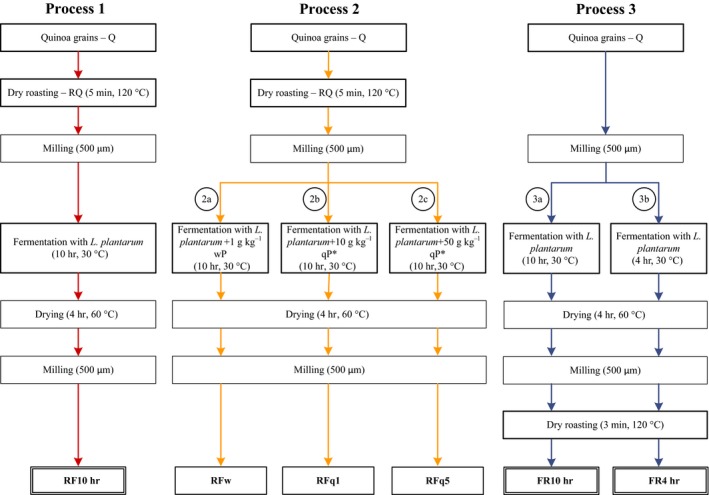
Description of quinoa processing. The processes included dry roasting and fermentation with *Lactobacillus plantarum*. Process 1: Dry‐roasted quinoa grains, milled, and fermented for 10 hr—RF10h. Process 2a: Dry‐roasted quinoa grains, milled, and fermented for 10 hr with addition of 1 g/kg wheat phytase—RFw. Process 2b: Dry‐roasted quinoa grains, milled, and fermented for 10 hr with addition of 10 g/kg activated quinoa phytase—RFq1. Process 2c: Dry‐roasted quinoa grains, milled, and fermented for 10 hr with addition of 50 g/kg activated quinoa phytase—RFq5. Process 3a: Raw quinoa flour fermented for 10 hr followed by dry roasting at 120°C for 3 min—FR10h. Process 3b: Raw quinoa flour fermented for 4 hr followed by dry roasting at 120°C for 3 min—FR4h. *The activated quinoa phytase (qP) was prepared mixing quinoa flour and water. This blend was kept at 30°C for 2 hr. The fermented flours used for hedonic sensory evaluation are within double‐line boxes

Figure 1 describes all fermentation processes. Fermentation was conducted in a suspension of quinoa flour and demineralized water (ratio 1:2 w/V), inoculated with *L. plantarum* 299v^®^ (7.35 Log_10_ CFU/g expressed in dry matter (DM)), and fermented at 30°C (Oven Termaks TS 8056) for 10 hr or 4 hr. Thereafter, the samples were dried in an oven (Termaks) for 4 hr at 60°C. The dried and milled samples (500 μm, Laboratory Mill 120; Perten Instruments AB) were stored in plastic bags at 4°C for analysis of iron, zinc, calcium, and phytate content. The following variations were included in each process: In process 1, quinoa grains were dry‐roasted (section 2.3) and milled prior to preparation of the suspension and fermented for 10 hr. In process 2a, the suspension prepared with dry‐roasted and milled quinoa grains was mixed with 1 g/kg wheat phytase (Sigma‐Aldrich) and fermented for 10 hr. In processes 2b and 2c, a back‐slop starter (10 g/kg and 50 g/kg, respectively) was included in order to add activated quinoa phytase to the suspension of dry‐roasted and milled quinoa grains, and the fermentation time was 10 hr. The back‐slop starter, called activated quinoa phytase (qP) in Figure 1, was prepared by mixing raw quinoa flour and demineralized water. This blend was kept at 30°C for 2 hr for activation of endogenous quinoa phytase (Castro‐Alba et al., 2019). In processes 3a and 3b, the substrate was raw quinoa flour and fermentation times were 10 hr and 4 hr, respectively. After fermentation, the samples were dried in an oven (Termaks) as described above and were then dry‐roasted as described in section 2.3.

### 2.3 Dry roasting of quinoa grains and flours

Whole quinoa grains were dry‐roasted in a pan (stainless steel pan, diameter 16 cm, Ikea, Sweden) on a stove (Electro (EH) Helios, induction stove, Electrolux) at 120°C for 5 min. The roasted grains were milled and sifted through a 500‐μm sieve (Perten Instruments AB). Fermented quinoa flour was dry‐roasted at 120°C for 3 min (stainless steel tray, 40*20 cm, Oven Termaks). The roasting times were based on local recipes, until development of an aroma and a brownish color.

### 2.4 Analytical procedures

#### 2.4.1 Moisture determination

The moisture content was determined by measuring water loss after drying 5 g (±0.0001 g) of each sample. Samples were dried at 105°C (heating oven; model ED23, Binder) until constant weight (AOAC, 2000).

#### 2.4.2 pH and acidity determination

pH and acidity were determined by duplicate according to Nuobariene et al. (2015) Ten grams of sample was mixed with 90 ml of deionized water and homogenized for 120 s. The pH values were measured with a pH meter (Denver Instrument, VB‐10 Ultra Basic). The acidity expressed as percentage of lactic acid was measured by titration with 0.1 N sodium hydroxide using phenolphthalein as indicator, until a faint pink color persisted for 30 s (AOAC, 2000).

#### 2.4.3 Mineral content determination

Iron, zinc, and calcium content were determined following the procedure described by Lazarte, Carlsson, et al. (2015)) Minerals were analyzed by flame atomic absorption spectrophotometry with air–acetylene flame (Model AAnalyst 200; PerkinElmer Corp.) at 248.3 nm for iron, 213.9 nm for zinc, and 422.7 nm for calcium.

#### 2.4.4 Phytate content determination and estimation of mineral bioavailability

Phytate content was determined using high‐performance ion chromatography (HPIC) method (Carlsson, Bergman, Skoglund, Hasselblad, & Sandberg, 2001; Lazarte, Carlsson, et al., 2015).

Mineral bioavailability was estimated using phytate:mineral molar ratios before and after dry roasting and fermentation with *L.* *plantarum* in each process. Phy:Fe, Phy:Zn, Phy:Ca, and Phy·Ca:Zn molar ratios were calculated with 660 g/mol as the molecular weight of phytate. The calculated molar ratios were compared with the suggested molar ratios of phytate:iron (Phy:Fe) <1; phytate:zinc (Phy:Zn) <15; phytate·calcium:zinc (Phy·Ca:Zn) <200; and phytate:calcium (Phy:Ca) <0.17 for adequate bioavailability of these minerals (Brown et al., 2004; Sandberg & Svanberg, 1991).

### 2.5 Viable count of lactobacilli

Fermented quinoa suspensions (1.0 g) were 10‐fold diluted with peptone solution (8.5 g/L NaCl, 1.0 g peptone/L). An aliquot (100 μl) was plated on Rogosa agar for lactobacilli count. The plates were incubated anaerobically at 37°C for 72 hr. The viable colonies were counted and expressed as Log_10_ CFU/g DM fermented quinoa (Håkansson et al., 2012).

### 2.6 Hedonic sensory evaluation of fermented quinoa flour

The hedonic sensory evaluation was conducted for fermented quinoa flours that achieved high phytate degradation. In a previous sensory evaluation with a small panel, non‐dry‐roasted fermented flour was tested and its acceptability was very low. Therefore, fermented flours were roasted before and after fermentation with the aim of improving the taste and reducing off‐taste. The treated samples were compared with nonfermented dry‐roasted quinoa flour, as a reference, on sensory characteristics. To assess the sensory attributes, porridge was prepared with fermented flours from processes 1 (dry‐roasted quinoa grains, milled, fermented for 10 hr—RF10h), 3a (raw quinoa flour fermented for 10 hr followed by dry roasting—FR10h), and 3b (raw quinoa flour fermented for 4 hr followed by dry roasting—FR4h) and nonfermented dry‐roasted quinoa flour (Rqf) following a traditional local recipe. Briefly, 100 g of flour was mixed with 150 ml of commercial cold lactose‐free milk avoiding formation of lumps. This blend was added to 650 ml of boiling lactose‐free milk (~92°C, Electrolux) and boiled under continuous stirring for 8 min until the porridge thickened.

The hedonic sensory evaluation of nonfermented and fermented quinoa flour porridges (RF10h, FR10h, and FR4h) was conducted by 35 untrained panelists, who were familiar with quinoa taste, recruited from San Simon University, Cochabamba, Bolivia. Before the sensory evaluation, each panelist was informed that the porridges were prepared with quinoa flour and lactose‐free milk. The porridges (40 ± 5 g) were served at 40 ± 10°C in transparent plastic cups with lids. The samples were served in randomized order during the session. The attributes used to evaluate the porridge quality were color, odor/aroma, taste, aftertaste, texture, and overall acceptability. The panelists graded the characteristics using a seven‐point hedonic scale (1—dislike extremely to 7—like extremely) (Meilgaard, Vance Civille, & Carr, 1999). Water at room temperature was provided to rinse the mouth between samples.

### 2.7 Statistical analysis

The results are presented as mean and standard deviation. The significance of fermentation for phytate and mineral content was tested using one‐way ANOVA of SPSS Statistics 24 (SPSS Inc., IBM Corporation). The Unscrambler^®^ X 10.2 software (CAMO Software AS) was used to perform a principal component analysis (PCA) to visualize the relationship between sensory attributes, analytical variables, and processing variables of quinoa. The PCA was performed with the results shown in Tables 1 and 3. All the data were centered and normalized.

## 3 RESULTS

The effects of dry roasting and *L. plantarum* 299v^®^ fermentation of quinoa after processes 1, 2, and 3 on pH, lactic acid, and phytate content are shown in Table 1. All processes had a significant effect (*p* < .05) on decreasing pH and increasing lactic acid content in quinoa flour. There were no significant differences in mineral content after the different processes (data not shown).

**Table 1:** Effect of dry roasting and lactic acid fermentation with *Lactobacillus plantarum* 299v^®^ on pH, acidity, cell viability, and phytate content in raw, dry‐roasted, and fermented quinoa (processes 1, 2, and 3)^1^, mean ± *SD* expressed in dry matter

Process	Sample	Moisture (g/kg)	Cell viability (Log_10_ CFU/g)^2^	pH	Lactic acid (g/kg)	Phytate (g/kg)	Phytate reduction (%)^3^
Process 1	Q	110 ± 0.06^b^	–	6.45 ± 0.01^b^	7.73 ± 0.06^a^	8.93 ± 0.25^c^	–
	RQ^4^	36.3 ± 2.5^a^	–	6.49 ± 0.01^b^	8.73 ± 0.12^b^	7.06 ± 0.20^b^	21.0
	RF10h^4^	44.7 ± 10^a^	9.47 ± 0.02	4.22 ± 0.04^a^	37.8 ± 0.69^c^	6.28 ± 0.20^a^	30.0
Process 2	Q	101 ± 0.28^e^	–	6.69 ± 0.02^e^	9.85 ± 0.12^a^	8.30 ± 0.50^c^	–
	RQ	47.3 ± 5.8^b^	–	6.59 ± 0.03^d^	10.1 ± 0.55^a^	6.78 ± 0.49^b^	19.2
2 a	RFw	73.3 ± 6.5^d^	–	4.36 ± 0.03^c^	30.4 ± 1.1^b^	5.32 ± 0.18^a^	35.9
2 b	RFq1	63.7 ± 1.8^c^	10.0 ± 0.14	4.24 ± 0.03^b^	40.4 ± 0.77^c^	5.62 ± 0.30^a^	32.3
2 c	RFq5	39.4 ± 4.9^a^	10.1 ± 0.21	4.12 ± 0.02^a^	39.5 ± 0.27^c^	5.03 ± 0.21^a^	39.4
Process 3	Q	99.0 ± 0.23^c^	–	6.71 ± 0.02^c^	12.0 ± 0.05^a^	7.92 ± 0.45^b^	–
3 a	FQ10h	32.6 ± 1.1^a^	–	4.28 ± 0.10^a^	46.4 ± 5.1^c^	2.14 ± 0.20^a^	73.0
3 a	FR10h	28.7 ± 0.49^a^	–	4.27 ± 0.10^a^	48.0 ± 5.3^c^	2.14 ± 0.08^a^	73.0
3 b	FQ4h	60.5 ± 3.8^b^	8.29 ± 0.07	4.91 ± 0.13^b^	38.4 ± 1.4^b^	2.20 ± 0.15^a^	72.0
3 b	FR4h	31.2 ± 4.0^a^	–	4.89 ± 0.14^b^	39.8 ± 1.7^b^	2.20 ± 0.13^a^	72.0

Note

Q: raw quinoa grains. RQ: dry‐roasted and milled quinoa grains. RF10h: dry‐roasted quinoa grains, milled, and fermented for 10 hr. RFw: dry‐roasted quinoa grains, milled, and fermented for 10 hr with addition of 1 g/kg wheat phytase. RFq1: dry‐roasted quinoa grains, milled, and fermented for 10 hr with addition of 10 g/kg activated quinoa phytase. RFq5: dry‐roasted quinoa grains, milled, and fermented for 10 hr with addition of 50 g/kg activated quinoa phytase. FQ10h: raw quinoa flour fermented for 10 hr. FR10h: raw quinoa flour fermented for 10 hr followed by dry roasting at 120°C for 3 min. FQ4h: raw quinoa flour fermented for 4 hr. FR4h: raw quinoa flour fermented for 4 hr followed by dry roasting at 120°C for 3 min.

^1^ Different letters in each parameter for each process indicate significant differences at *p* < .05

^2^ Growth of lactobacilli in fermented quinoa before drying at 60°C. At the beginning of the process, 7.35 Log_10_ CFU/g DM were added.

^3^ Phytate content degradation from raw quinoa.

^4^ The results of pH, lactic acid, and phytate have been previously reported Castro‐Alba et al. (2019).

The phytate degradation varied between 30% and 73% from initial content in raw quinoa and depended on type of quinoa substrate (dry‐roasted or raw flour) and source of phytase. It was found that the phytate degradation in dry‐roasted quinoa grains, milled, followed by fermentation (process 1) was significantly lower (*p* < .05) than after fermentation of dry‐roasted and milled quinoa grains with 1 g/kg wheat phytase (process 2a) or with 50 g/kg activated quinoa phytase (process 2c). Regarding the effect of addition of exogenous phytase on phytate degradation in process 2, there was no significant difference between addition of 1 g/kg wheat phytase (process 2a) or 50 g/kg activated quinoa phytase (process 2c). However, addition of 50 g/kg activated quinoa phytase resulted in a significantly higher (*p* < .05) phytate degradation than addition of 10 g/kg activated quinoa phytase (process 2b). In process 3, fermentation of raw quinoa flour for 10 hr (process 3a) had the same effect on phytate degradation as fermentation for 4 hr (process 3b). Regarding the effect of dry roasting before and after fermentation, it was found that the degradation of phytate in process 3 (73%) where dry roasting was conducted after fermentation was significantly (*p* < .05) higher than that of process 1 (30%) where dry roasting was performed prior to fermentation.

The Phy:Fe, Phy:Zn, Phy:Ca, and Phy·Ca:Zn molar ratios shown in Table 2 were calculated for the fermentation process 3 that achieved a higher degradation of phytate. The molar ratios of all studied minerals were significantly reduced (*p* < .05). Phy:Zn and Phy·Ca:Zn were reduced below the critical value, which indicated that the zinc estimated bioavailability was improved from low to moderate.

**Table 2:** Effect of lactic acid fermentation with *Lactobacillus plantarum* 299v^®^ on mineral content and their estimated bioavailability in raw, fermented, and dry‐roasted fermented quinoa flour, mean ± *SD* expressed in dry mater

Process	Samples	Iron mg/kg	Zinc mg/kg	Calcium mg/kg	Phy:Fe	Phy:Zn	Phy:Ca	Phy·Ca:Zn
Process 3	Q	52.3 ± 2.0	30.8 ± 0.20	465 ± 36	12.8 ± 0.96^b^	25.4 ± 1.4^b^	1.04 ± 0.11^b^	295 ± 30^b^
3 a	FQ10h	50.2 ± 1.5	32.8 ± 0.20	490 ± 34	3.60 ± 0.44^a^	6.44 ± 0.60^a^	0.27 ± 0.03^a^	79.0 ± 10^a^
3 a	FR10h	48.0 ± 2.1	32.3 ± 1.5	439 ± 62	3.79 ± 0.28^a^	6.57 ± 0.48^a^	0.26 ± 0.01^a^	82.3 ± 10^a^
3 b	FQ4h	49.3 ± 1.0	30.0 ± 0.50	457 ± 32	3.78 ± 0.28^a^	7.26 ± 0.46^a^	0.31 ± 0.06^a^	79.2 ± 10^a^
3 b	FR4h	47.0 ± 2.2	30.5 ± 0.40	464 ± 44	3.96 ± 0.27^a^	7.14 ± 0.38^a^	0.29 ± 0.02^a^	81.5 ± 8.2^a^

Note

Q: raw quinoa grains. FQ10h: raw quinoa flour fermented for 10 hr. FR10h: raw quinoa flour fermented for 10 hr followed by dry roasting at 120°C for 3 min. FQ4h: raw quinoa flour fermented for 4 hr. FR4h: raw quinoa flour fermented for 4 hr followed by dry roasting at 120°C for 3 min.

^1^ Different letters in each parameter for each process indicate significant differences at *p* < .05.

The viable counts of lactobacilli after fermentation are shown in Table 1. The cell growth in processes 1, 2b, and 2c was higher than in process 3b due to the longer fermentation time. The addition of back‐slop starter to dry‐roasted and milled quinoa grain suspensions (processes 2b and 2c) contributed to higher cell counts, probably due to the cell count in these suspensions being from a mixed lactobacilli microbiota (*L.* *plantarum* and endogenous microorganisms).

The results of the hedonic sensory evaluation of nonfermented and fermented quinoa flour are shown in Table 3. The results showed that there was no significant difference in the overall acceptability of porridges made with nonfermented quinoa flour and fermented flours from processes 1 (dry‐roasted quinoa grains, milled followed by fermentation for 10 hr) and 3b (fermentation of raw flour for 4 hr followed by dry roasting). The reduction of fermentation time for raw quinoa flour from 10 hr to 4 hr (processes 3a and 3b) had a positive significant effect (*p* < .05) on the overall acceptability.

**Table 3:** Sensory evaluation^1^ of nonfermented and fermented quinoa flour as porridge^2^, mean ± standard deviation

Process	Sample	Color	Odor/Aroma	Taste	Aftertaste	Texture	Overall acceptability
‐	Rqf	3.97 ± 1.4^a^	3.31 ± 1.5^a^	4.00 ± 1.6^b^	3.89 ± 1.4^b^	3.71 ± 1.4^a^	3.91 ± 1.7^b^
1	RF10h	4.69 ± 1.4^ab^	4.40 ± 1.4^b^	4.03 ± 1.4^b^	3.94 ± 1.4^b^	4.69 ± 1.4^b^	4.20 ± 1.3^b^
3a	FR10h	4.86 ± 0.81^b^	3.74 ± 1.2^ab^	2.29 ± 0.89^a^	2.57 ± 0.98^a^	5.06 ± 0.91^b^	3.03 ± 1.0^a^
3b	FR4h	4.74 ± 1.2^b^	3.74 ± 1.5^ab^	4.14 ± 1.5^b^	4.40 ± 1.3^b^	5.26 ± 0.85^b^	4.51 ± 1.4^b^

Note

Rqf: nonfermented dry‐roasted quinoa flour. RF10h: dry‐roasted quinoa grains, milled, and fermented for 10 hr. FR10h: raw quinoa flour fermented for 10 hr followed by dry roasting at 120°C for 3 min. FR4h: raw quinoa flour fermented for 4 hr followed by dry roasting at 120°C for 3 min.

^1^ A seven‐point hedonic scale (1—dislike extremely to 7—like extremely) was used in sensory evaluation.

^2^ Different letters in each parameter indicate significant differences at *p* < .05.

**Figure 2:**
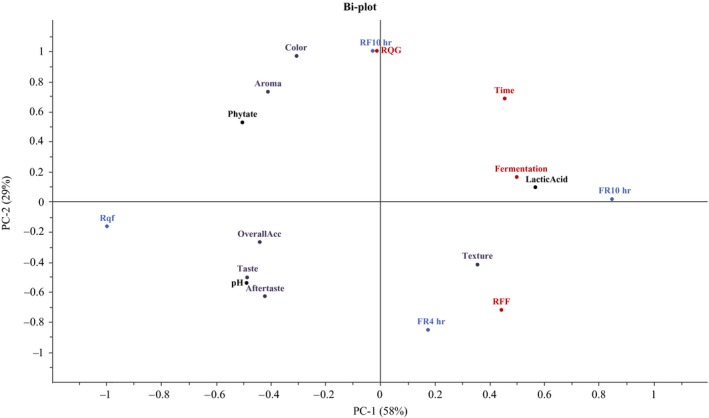
Principal component analysis biplot from four porridges prepared with fermented quinoa flour from processes 1 (RF10h) and 3 (FR10h, FR4h) and nonfermented flour (Rqf) and 13 variables which include six sensory attributes, three analytical variables, and four processing variables. Results are expressed in dry matter. Rqf: nonfermented dry‐roasted quinoa flour. RF10h: dry‐roasted quinoa grains, milled, and fermented for 10 hr. FR10h: raw quinoa flour fermented for 10 hr followed by dry roasting at 120°C for 3 min. FR4h: raw quinoa flour fermented for 4 hr followed by dry roasting at 120°C for 3 min. RQG: dry roasting of raw grains. RFF: dry roasting of fermented flour

The PCA biplot for the four tested porridges (Figure 2) shows the relationship between sensory attributes, analytical (pH, lactic acid content, and phytate) and processing variables RQG (dry roasting of raw grains), fermentation, time, and RFF (dry roasting of fermented flour)). Two principal components accounted for 87% of the variance in six sensory attributes, three analytical variables, and four processing variables. The principal component 1 (PC1 = 58%) was explained by four sensory attributes (taste, aftertaste, texture, and overall acceptability), all the analytical variables (pH, lactic acid, and phytate), and three processing variables (fermentation, time, and RFF). The principal component 2 (PC2 = 29%) explained the variations in sensory attributes (color and aroma) and one processing variable (RQG). PC1 was positively correlated with lactic acid (0.99), fermentation (0.87), time (0.79), RFF (0.77), and texture (0.62), while negative correlations were found for phytate (−0.88), pH (−0.86), taste (−0.85), overall acceptability (−0.77), and aftertaste (−0.74). PC2 was positively correlated with RQG (0.87), color (0.84), and aroma (0.64).

Regarding the relationship between analytical variables and sensory attributes, Figure 2 shows that pH was positively related to taste, aftertaste, and overall acceptability of fermented quinoa flour porridges and lactic acid content had a negative relationship with these attributes. Lactic acid content had a positive effect on the texture of the porridges, but a negative effect on the overall acceptability. Regarding overall acceptability, it made no difference whether the dry roasting was performed on the grains before fermentation (RQG) or on the fermented quinoa flour (RFF). However, the dry roasting processing (RQG) showed a positive correlation with the color and aroma of porridges.

## 4 DISCUSSION

The present study showed that fermentation and dry roasting significantly reduced phytate content in quinoa. Additionally, dry roasting had a positive effect on improving sensory properties. The results indicated that phytate content was reduced during fermentation to levels that improved the estimated bioavailability of iron and zinc. The sensory properties of the quinoa flour fermented for 4 hr followed by dry roasting were comparable to those of nonfermented dry‐roasted quinoa flour, which was used as reference of adequate overall acceptability.

Fermentation is an effective method of reducing phytate content in quinoa flour. At the end of process 1, fermentation of dry‐roasted and milled quinoa grains, phytate was degraded by 30% from the initial content. This degradation might be mainly due to exogenous phytase activity of *L.* *plantarum* rather than endogenous phytase activity, but it seems that *L.* *plantarum* has a weak phytase activity at pH range 4.22 – 6.49. In process 2, the addition of wheat phytase 1 g/kg (2a) or activated quinoa phytase 10 g/kg (2b) and 50 g/kg (2c) to the suspensions, made of dry‐roasted and milled quinoa grains, increased phytate degradation to 36%, 32%, and 39%, respectively. This relatively low degradation might be explained by the fact that the optimal pH of 4.5–5.0 for phytase activity( Sandberg & Svanberg, 1991) was maintained only for a short time due to the further drop in pH during fermentation. In addition, the fermentation was conducted at 30°C while the optimum temperature for phytase activity is reported to be 50°C (Sandberg & Svanberg, 1991). During process 3, fermentation of raw quinoa flour for 10 hr (3a) or 4 hr (3b) followed by dry roasting, phytate was degraded between 1.8‐fold and 2.2‐fold more than when dry roasting of grains was conducted before fermentation (processes 1 and 2). This higher degradation was mainly due to the activation of endogenous phytase of quinoa flour (Castro‐Alba et al., 2019). Valencia et al., (1999) and Dallagnol, Pescuma, De Valdez, and Rollán (2013) reported a similar degradation of phytate content after fermentation with different strains of *L.* *plantarum*.

Dry roasting had to some extent a positive effect on the degradation of phytate in quinoa. The dry roasting of quinoa grains before fermentation (processes 1 and 2) degraded phytate content by 20% from initial levels. It is likely that the phytate in the quinoa grains was degraded by their endogenous phytase, which was activated by a gradual increase in temperature during the early stage of the dry roasting treatment. According to Greiner and Konietzny (1998) a temperature above 65°C may inactivate endogenous phytase, after which no further phytate degradation can be expected. In this regard, Brejnholt, Dionisio, Glitsoe, Skov, and Brinch‐Pedersen (2011) reported that endogenous phytase activity of wheat was reduced by 93% after a heat treatment (95°C, 10 min). The thermal treatment in our study was carried out at 120°C; it is therefore likely that at the end of this treatment, the endogenous phytase of the quinoa grains was no longer active. The effect of dry roasting on phytate content in fermented quinoa flour (processes 3a and 3b) was negligible; this may be due to the fact that fermented suspension was dried at 60°C before dry roasting, a temperature that may have inactivated any remaining phytase activity after fermentation of the raw quinoa flour.

The different processes had significant effects on the reduction of molar ratios and, therefore, the improvement of estimated bioavailability. The reduction of phytate when dry roasting was performed before fermentation (processes 1 and 2) was not enough to decrease Phy:Fe, Phy:Zn, Phy:Ca, and Phy·Ca:Zn molar ratios under the critical values for an improved estimated bioavailability of iron, zinc, and calcium. Fermentation of raw quinoa flour followed by dry roasting (processes 3a and 3b) improved the estimated zinc bioavailability from low to moderate with molar ratios below the threshold for Phy:Zn (7.14) and Phy·Zn:Ca (81.5). The values for Phy:Fe (3.96) and Phy:Ca (0.29) molar ratios were significantly reduced, but still above threshold for improved bioavailability of iron and calcium. It has been reported that to reduce Phy:Fe molar ratios below threshold, phytate in wheat should be reduced between 95% and 100% (Sandberg & Svanberg, 1991). In the present study, in order to reduce Phy:Fe molar ratios below threshold, phytate in quinoa should be reduced by at least 93% from initial content. To decrease Phy:Ca below the critical value, phytate should be degraded by at least 85% from initial content.

It is known that lactic acid fermentation of wholemeal flours can improve the appearance and flavour (Poutanen, Flander, & Katina, 2009; Salmeron, 2017). However, it is also known that off‐flavor compounds can be produced during fermentation (Di Renzo et al., 2018). The modification of flavor is based on the production of amino acids, small peptides, and phenolic compounds released during fermentation by the metabolism of microorganisms (Thiele, Gänzle, & Vogel, 2002) as well as the production of sugars and organic acids, which can contribute to the sour taste of foods (Salmeron, Thomas, & Pandiella, 2015). In our study, the type of quinoa substrate (dry‐roasted or raw flour) and fermentation time contributed to the growth of lactobacilli and the production of organic acids, mainly lactic acid (Table 1). Fermentation of milled dry‐roasted quinoa grains (processes 1 and 2) produced between 20% and 27% less lactic acid than fermentation of raw quinoa flour for the same period of time (process 3a). The reduction of the fermentation time for raw quinoa flour from 10 hr (process 3a) to 4 hr (process 3b) resulted in a decrease in lactic acid production of approximately 20%. Although volatile compounds were not analyzed in the current study Di Renzo et al. (2018) reported the production of 49 volatile compounds during fermentation of quinoa flour dough. These compounds belonged mainly to aldehydes, sulfur compounds, ketones, ester and acetate, alcohols, furans, pyrazines, and acids. Dallagnol et al. (2013) reported that lactic acid fermentation stimulated flour protein hydrolysis by endogenous proteases of quinoa. One of the by‐products of protein hydrolysis is dimethyl sulfide, which in low concentrations confers aromatic flavor to food, but at high levels has a characteristic disagreeable odor commonly described as cabbage‐like (Di Renzo et al., 2018).

Dry roasting is a suitable process for developing flavor and color compounds in foods through the Maillard reaction, in which amino compounds react with reducing sugars, and caramelization reactions, which occur between sugars (Fayle et al., 2002). The Maillard reaction depends on the type of substrate, temperature, time, water activity, and pH (Ramírez‐Jiménez, García‐Villanova, & Guerra‐Hernández, 2001). The dry roasting of quinoa grains before fermentation (processes 1 and 2) was carried out at 120°C to develop flavor compounds and to reduce off‐flavors during fermentation. Similarly, fermented raw flours (process 3) were dry‐roasted to evaporate volatile off‐flavor compounds and produce more flavor compounds. It was reported that temperatures below 140°C favor the Maillard reaction; (Rufián‐Henares, Delgado‐Andrade, & Morales, 2009) consequently, Carciochi (2016) indicated that roasting of quinoa grains at 130°C resulted in the formation of brown polymers. In our study, the dry roasting process of whole quinoa grains required more time (5 min) to develop flavor and brown color than fermented flour (3 min). This difference in time may be due to the fact that fermented quinoa flour had higher amounts of free amino acids and sugars, which favor the Maillard reaction (Dallagnol et al., 2013). In addition, whole grains require more time than flour to evaporate water and reach the temperature for formation of Maillard reaction compounds (Lingnert, 1990). pHs of grains (6.44–6.70) and fermented quinoa flours (4.28–4.91) were in the suitable pH range (4–7) for formation of 5‐hydroxymethylfurfural (HMF), which is the precursor for formation of melanoidins, the brown polymers (Parisi & Luo, 2018) Flavor compounds such as aldehydes, pyrazines, pyrroles, and furfurals, typical of toasted cereals, are also formed in this pH range (Bamforth & Bamforth, 2007). Other compounds formed during the Maillard reaction are alkylpirazines, alkylpyridines (regarded as unpleasant), acylpyridines, furans, furanones, and pyranones (Van Boekel, 2006).

**Figure 3:**
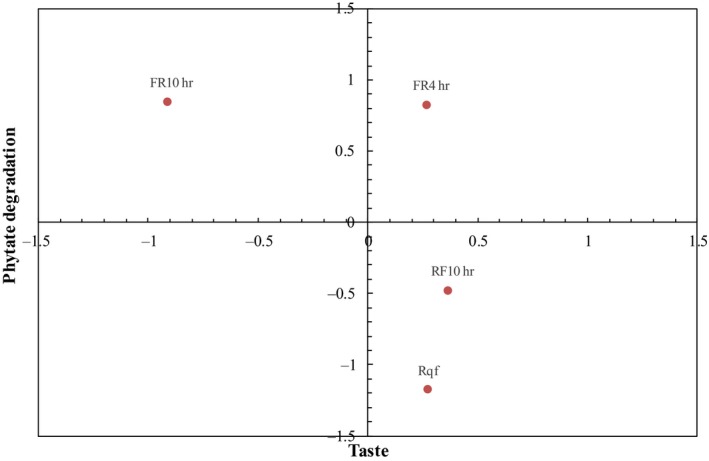
Relation between taste and phytate degradation for four porridges prepared with fermented quinoa flour from processes 1, dry‐roasted quinoa grains, milled, and fermented for 10 hr (RF10h), and process 3, raw quinoa flour fermented for 10 hr or 4h followed by dry roasting (FR10h, FR4h) and nonfermented dry‐roasted flour (Rqf). All data were normalized and centered

A sensory analysis was conducted to evaluate the acceptability of fermented quinoa flours, prepared as porridges. In a prior sensory evaluation (with a small panel of six people), porridges prepared with non‐dry‐roasted fermented quinoa flour were tested, and the results showed that the acceptance of the product was very low (data not shown). Therefore, dry roasting was used as an alternative to improve the sensory characteristics of the fermented flour. The results of the sensory analysis presented in this paper showed that the overall acceptability of porridge made with raw quinoa flour fermented for 4 hr followed by dry roasting (process 3b) was comparable to porridge made with dry‐roasted and milled quinoa grains followed by fermentation (process 1) and porridge made with nonfermented dry‐roasted quinoa flour. The acceptability of these three flours was significantly higher than that of porridge made with raw quinoa flour fermented for 10 hr followed by dry roasting (process 3a). According to PCA, the overall acceptability of quinoa flours was strongly influenced by their taste and aftertaste attributes (Figure 2). Both sensory attributes were affected by the increased lactic acid content and pH drop of flours; the changes in both analytical parameters resulted from the fermentation time and type of substrate, which also had a strong impact on phytate degradation (Table 1). Figure 3 shows the relation between taste and phytate degradation for the different flours prepared as porridges. Two porridges were prepared with flours with high degradation of phytate (process 3, fermented for 4 hr and 10 hr). Between these two, the porridge prepared with raw quinoa flour fermented for 4 hr had a better taste, which was comparable to the taste of porridges prepared with flours from process 1 and with nonfermented dry‐roasted quinoa flour, although these last two porridges had higher phytate content. It was reported that fermented beverages with mild acidic pH values (3.5–4.5) have higher consumer acceptability (Chun, Kwon, Kim, & Kim, 2008; Salmeron et al., 2015). Regarding aroma, the most preferred porridge was prepared with dry‐roasted quinoa grains, milled followed by fermentation. Msheliza, Ilesanmi, Hussein, and Nkama (2018) have reported that roasting and fermentation followed by roasting of sorghum and soy improved the acceptability of gruel prepared with blends of both flours. In the present study, the dry roasting process of grains before fermentation or of fermented flour had the same positive effect on the overall acceptability of the final product. Taking these considerations into account, we found that fermentation of raw quinoa flour for 4 hr followed by dry roasting was the optimal process to obtain fermented flour with good sensory properties and the highest phytate degradation. It was then achieved an adequate Phy:Zn molar ratio indicating an improved estimated zinc bioavailability.

## 5 CONCLUSION

Nutritional and sensory attributes are important characteristics for acceptability of fermented products. Fermentation improves nutritional properties of food and may produce flavor‐enhancing compounds, but it also has the potential to produce disagreeable off‐flavor compounds, and it may therefore become a challenge to obtain final products with acceptable sensory properties. This study shows that dry roasting of quinoa grains before fermentation or dry roasting of fermented flour improved the sensory attributes and overall acceptability of the final product. However, dry roasting before fermentation inactivated endogenous phytase, thus resulting in a low degradation of phytate even if wheat phytase or activated quinoa phytase were added during processing. Fermentation of raw quinoa flour (for 4 hr or 10 hr) was very effective in degrading phytate to levels that improved the estimated zinc bioavailability in fermented quinoa flour. However, 10‐hr fermentation produced a higher level of lactic acid, which decreased the acceptability of the fermented flour. Taking into account the degradation of phytate, improvement of estimated bioavailability, and sensory attributes, it was found that fermentation of raw quinoa flour for 4 hr followed by dry roasting was successful in improving both nutritional and sensory attributes of the final product.

## CONFLICT OF INTEREST

The authors declare no conflict of interest.

## ETHICAL APPROVAL

The authors declare that human and animal testing was unnecessary in this study.
